# MicroRNA-Mediated Down-Regulation of M-CSF Receptor Contributes to Maturation of Mouse Monocyte-Derived Dendritic Cells

**DOI:** 10.3389/fimmu.2013.00353

**Published:** 2013-10-30

**Authors:** Joey Riepsaame, Adri van Oudenaren, Berlinda J. H. den Broeder, Wilfred F. J. van IJcken, Joris Pothof, Pieter J. M. Leenen

**Affiliations:** ^1^Department of Immunology, Erasmus University Medical Center, Rotterdam, Netherlands; ^2^Erasmus Center for Biomics, Erasmus University Medical Center, Rotterdam, Netherlands; ^3^Department of Genetics, Erasmus University Medical Center, Rotterdam, Netherlands

**Keywords:** mouse, dendritic cells, microRNAs, M-CSR receptor, *Csf1r*

## Abstract

Dendritic cell (DC) maturation is a tightly regulated process that requires coordinated and timed developmental cues. Here we investigate whether microRNAs are involved in this process. We identify microRNAs in mouse GM-CSF-generated, monocyte-related DC (GM-DC) that are differentially expressed during both spontaneous and LPS-induced maturation and characterize M-CSF receptor (M-CSFR), encoded by the *Csf1r* gene, as a key target for microRNA-mediated regulation in the final step toward mature DC. MicroRNA-22, -34a, and -155 are up-regulated in mature MHCII^hi^ CD86^hi^ DC and mediate *Csf1r* mRNA and protein down-regulation. Experimental inhibition of *Csf1r*-targeting microRNAs *in vitro* results not only in sustained high level M-CSFR protein expression but also in impaired DC maturation upon stimulation by LPS. Accordingly, over-expression of *Csf1r* in GM-DC inhibits terminal differentiation. Taken together, these results show that developmentally regulated microRNAs control *Csf1r* expression, supplementing previously identified mechanisms that regulate its transcription and protein surface expression. Furthermore, our data indicate a novel function for *Csf1r* in mouse monocyte-derived DC, showing that down-regulation of M-CSFR expression is essential for final DC maturation.

## Introduction

Dendritic cells (DC) constitute a heterogeneous population of leukocytes that interconnect the innate and the adaptive immune response, in particular through their capacity to activate naïve T lymphocytes (1). DC depend on several growth factors for their proliferation, survival, and differentiation, most importantly Flt3L, GM-CSF, and M-CSF (2). Flt3L drives the development of various DC populations, in particular plasmacytoid and conventional DC (cDC), in peripheral tissues and lymphoid organs in the steady-state (3), whereas GM-CSF is important in generating inflammatory, monocyte-derived TNF/iNOS-producing DC (TipDC) (1, 4). Inactivation of M-CSF or its receptor *in vivo* results in a significant decrease in DC numbers (5, 6) and shift in DC subset composition (7), including a complete absence of epidermal Langerhans cells (8) and monocyte-derived DC in the intestinal lamina propria (9). Interestingly, M-CSF has been shown to induce also plasmacytoid and cDC development, in addition to development of macrophages, from BM cells of normal and Flt3L-knock out mice (10, 11). These observations underline a critical role of M-CSF signaling in the development of several DC populations.

Dendritic cells can initiate various types of T-cell responses, depending in part on the developmental status of the DC interacting with the T cells. Immature DC (iDC) characterized in mice as CD11c^+^MHC class II^low^CD86^low^ cells, are specialized in taking up and processing antigens but are poor immune stimulators and may induce tolerance. In contrast, mature DC (mDC), characterized as CD11c^+^MHC class II^hi^CD86^hi^ cells, induce cell-mediated and/or humoral immune responses (12, 13). Thus, tight regulation of DC maturation is required to maintain a proper immune balance.

MicroRNAs are an important class of regulators involved in differentiation and cell fate decisions (14, 15). They represent an extensive family of short (∼22 nt) single-stranded non-coding RNAs that regulate gene expression at a post-transcriptional level by binding to the 3′untranslated region (3′UTR) of mRNAs, thereby causing translational inhibition of the target mRNA primarily as a result of mRNA degradation (16). In recent years, microRNAs have emerged as important regulators of immune function, which has been demonstrated in particular by *in vivo* gain- or loss-of-function microRNA studies (17, 18). Thus far, however, most studies linking microRNAs with the immune system have focused on T and B lymphocytes, while only a limited number of studies have focused on their role in DC development and function (19, 20). Studies using human cells have shown that the microRNA expression profiles alter during DC development (20–24). Manipulating microRNA expression affects DC function in both human and mouse (21, 25). Here, we approached the question whether microRNAs are involved in regulating mouse monocyte-derived DC maturation focusing on the final stages where CD11c^+^MHC class II^low^CD86^low^ iDC develop into CD11c^+^MHC class II^hi^CD86^hi^ mDC. We determined the microRNA expression profiles of different mouse GM-DC maturation stages during GM-CSF-stimulated development *in vitro*. A set of microRNAs is described, which expression is prominently up-regulated during both the spontaneous and lipopolysaccharide (LPS)-induced transition of iDC to mDC. *Csf1r*, the gene encoding the growth factor receptor M-CSFR (c-Fms, M-CSFR, CD115), is identified as a predominant common target regulated by the induced miR-22, miR-34a, and miR-155. Moreover, we show that down-regulation of M-CSFR expression is a prerequisite for final DC maturation.

## Materials and Methods

### Animals

Female C57BL/6J mice were obtained from Harlan (Horst, Netherlands) and were kept under specific pathogen-free conditions at the animal facility of the Erasmus MC, Rotterdam, Netherlands. Housing, care, and experimental handling were performed in accordance with Dutch legal regulations. Ethical approval was obtained after protocol review by the independent animal experiment committee DEC Consult, and registered under permit numbers EUR1408 (128-08-05), EUR1738 (128-09-02), EMC2135 (128-10-10), and EMC2759 (128-12-07).

### DC maturation *in vitro*

Monocyte-derived DC were generated by GM-CSF stimulation of bone marrow (BM) precursors as described previously (26). These cells are indicated as GM-DC. Briefly, BM cells isolated from 8 to 13-week-old C57BL/6 mice were cultured in RPMI-1640 medium (Lonza, Belgium) supplemented with 10% fetal calf serum, 2 mM glutamine, 100 U/ml penicillin, 100 μg/ml streptomycin, 50 μM 2-mercaptoethanol, and 20 ng/ml rmGM-CSF (Biosource International, Camarillo, CA, USA). Cells were kept in a humidified incubator at 37°C with 5% CO_2_. At day 0, BM leukocytes were seeded at 3 × 10^5^ per ml in either 100 mm dishes (BD Biosciences), 12-wells plates (Nunc) or 96-wells round-bottom plates (Nunc). At day 3, fresh culture medium was added to the plates and at day 6, half of the medium was replaced. To induce enforced GM-DC maturation, 100 ng/ml LPS (*Escherichia coli* strain 055:B5, Sigma) was added on day 6. Alternatively, plasmacytoid DC (pDC) and cDC were generated by Flt3L stimulation of BM precursors essentially as described by Naik *et al*. with minor modifications (27). To this end, BM cells isolated from 8 to 13-week-old female C57BL/6 mice and erythrocytes were lysed by treatment for 2 min with 0.155 M NH_4_Cl. Then, BM cells were extensively washed and cultured in RPMI-1640 (Lonza, Belgium) supplemented with 10% fetal calf serum, 2 mM glutamine, 100 U/ml penicillin, 100 μg/ml streptomycin, 50 μM 2-mercaptoethanol, and 200 ng/ml Flt3L (Peprotech, Rocky Hill, NJ, USA), and seeded at 4 × 10^6^ per 2 ml in six-wells plates (Nunc). At day 7, cells were washed to remove free Flt3L, and stimulated for 24 h with 10 μg/ml CpG (ODN 2395, InvivoGen, San Diego, CA, USA).

To assess the role of TNF-α converting enzyme (TACE) in M-CSFR down-regulation, TACE inhibitors TMI-1 and TMI-2 (28, 29) were used in a final concentration of 10 and 15 μM, respectively (kindly provided by Dr. B. Scholte, Erasmus, MC, Netherlands). The functional inhibition during overnight cultures was tested by determining the TACE-mediated decrease of M-CSFR expression on BM monocytes. To that end, freshly isolated BM cells (10^6^ per ml) were cultured at 37°C for 24 h in a 12-well plate in RPMI-1640 with 10% FCS and antibiotics as described before, but without additional growth factors. Inhibitors were added from the start of the culture. As TACE inducer *E. coli* LPS (O55 B5, Sigma) was used in a final concentration of 100 ng/ml. Cells were harvested after 24 h. Similarly, TACE activity was inhibited in GM-DC cultures by adding inhibitors during the last 24 h of a 7-day culture in combination, either or not in the presence of LPS. Expression of M-CSFR/CD115 was determined as described below.

### Flow cytometry and cell sorting

For cell labeling, incubations were performed in staining buffer (PBS pH 7.8, 1% BSA, 0.01% sodium azide) on ice for 30 min. Reagents used were fluorescent conjugates of CD11b (M1/70), CD11c (HL3), CD86 (GL1), CD115 (anti-M-CSFR, clone AFS98), mMGL/CD301 (ER-MP23), MHC class II I-A/I-E (M5/114.15.2), SiglecH (eBio440c), and rat-anti-mouse IgG-Alexa488 and streptavidin-Alexa633. These antibodies were obtained from BD Biosciences, eBioscience, Molecular Probes or prepared as purified Ig from hybridomas created in our lab. Cells were analyzed by flow cytometry using a FACSCalibur or FACSCanto II (Becton Dickinson) and FlowJo Analysis Software (Tree Star, Ashland, OR, USA). Sorting of cells was performed using a FACSAria Cell Sorter (Becton Dickinson).

### MicroRNA microarray hybridization and analysis

Total RNA was extracted using acid-phenol:chloroform (Ambion) extraction and enriched for microRNAs using a mirVana microRNA isolation kit (Ambion) according to the manufacturer’s protocols. RNA was labeled using a ULS™ aRNA labeling kit (Kreatech Diagnostics, Amsterdam). 1.5 μg of total RNA was incubated with Cy3-ULS for 30 min at 85°C and purified to remove unbound Cy3-ULS. Labeled RNA was hybridized on miRCURY LNA microRNA arrays (probe set 8.0; Exiqon, Vedbaek, Denmark) at 60°C for 16 h using a Tecan 4800 hybridization station. Slides were washed and immediately scanned using a Tecan LS Reloaded microarray laser scanner. Microarray data extraction, normalization, and data analysis were carried out as described (30). Heatmaps were generated using the TM4 microarray software suite (31). Significance Analysis of Microarrays (SAM) analysis was carried out on sorted DC populations obtained from three biological replicates, implementing a false discovery rate (FDR) ≤ 10% and a minimum 1.5-fold change in expression. All data are MIAME compliant. Raw data have been deposited in ArrayExpress and are accessible under numbers A-MEXP-2085 and E-MEXP-3311.

### *Csf1r-*3′UTR luciferase reporter assay

The full-length *Csf1r-*3′UTR was cloned into the *Xho*I/*Not*I site downstream the coding sequence of *Renilla luciferase* in the psiCHECK-2 luciferase reporter vector (Promega). Cloning primers *Csf1r-*3′UTR: FW 5′-GGATTCCTCGAGTCCTGCCGCT-CTCTACGT-3′ and RE 5′-GGATTCGCGGCCGCCTGGCTGTGTTAATGCTGTT-AGTT-3′. Mutant *Csf1r-*3′UTR constructs were generated by introducing three basepair mismatches into each seed region of the corresponding miR-22, -34a, and -155 binding sites (*Csf1r*-3′UTR mut all) or the miR-22 site alone (*Csf1r*-3′UTR 22mut) [outsourced to Genscript (Piscataway, USA)]. HEK293T cells, described by Stewart et al. (32), were plated in a 48-well plate at a density of 6 × 10^4^ cells per well and then co-transfected the next day with 10 ng psiCHECK-2 vector containing the full 3′UTR of *Csf1r* mRNA, together with miR-22, -34a, -155, and control over-expression oligonucleotides (Ambion) at 50 nM final concentration using Lullaby transfection reagent (Boca Scientific). Luciferase activity was measured 48 h later using the Dual Glow luciferase kit (Promega) in a TopCount NXT microplate luminescence counter (Packard Instrument Company, Connecticut, USA). Transfections were performed in duplicate and repeated three times in independent experiments. Statistical analysis was by unpaired two-tailed Student’s *t* test; *p*-values of less than 0.05 were considered significant.

### Transient microRNA inhibition in GM-DC

3 × 10^5^ BM cells were cultured in 12-well plates to generate GM-DC. These cells were transfected on day 4 of culture with 50 nM anti-miR microRNA inhibitors (Ambion), mixed with siGLO Cy3-labeled non-targeting anti-miR oligonucleotides (Dharmacon) at a 5:1 ratio. Control samples were treated with a control non-targeting inhibitor (Dharmacon). Transient transfection was accomplished using DharmaFECT1 reagent (Dharmacon) according to the manufacturer’s protocol.

### Transient *Csf1r* over-expression in GM-DC

The complete ORF of mouse *Csf1r* was purchased as a full-length cDNA clone (Open Biosystems, IMAGE accession no. 30436119). GM-DC (8 × 10^6^ cells) were co-electroporated at day 6 of culture with 8 μg of the *Csf1r* cDNA clone and 2 μg pEGFP-C1 control vector (Clontech) using an Amaxa nucleofector apparatus (Lonza; program Y-01) and Amaxa mouse macrophage nucleofector kit (Lonza) according to the manufacturer’s instructions. For control experiments, cells were co-electroporated with 8 μg pCMV-SPORT6 vector and 2 μg pEGFP-C1 vector.

### Quantitative real-time PCR of microRNAs and *Csf1r*

The quantification of mature miR-155, -34a, and -22 expression levels was carried out with 1 μg of total RNA using the miScript PCR System (Qiagen) according to the manufacturer’s instructions. ΔCt values for each microRNA were normalized to tubulin reference gene. miR-155 FW primer: 5′-TTAATGCTAATTGTGATAGGGG-3′. miR-34a FW primer: 5′-TGGCAGTGTCTTAGCTGGTTGT-3′. miR-22 FW primer: 5′-AAGCTGCCAGTTGAAGAACTGT-3′. Tubulin primers: FW 5′-CAGACCAACCACT-GCTACAT-3′ and RE 5′-AGGGAATGAAGTTGGCCAGT-3′.

### *Ex vivo* analysis of M-CSFR expression

FITC painting of mice was performed as described previously (33). Briefly, mice were painted on the shaved back with 250 μl of 1% FITC (Sigma) in 1:1 acetone:dibutylphthalate (Sigma) and draining axillary and brachial lymph nodes (LN) were collected 24 h afterward. Mesenteric LN were taken as a control. Cells were isolated by mechanical disruption of the LN, without enzymatic treatment, and stained with CD301/mMGL (ER-MP23), CD11b, CD86, anti-MHC class II I-A/I-E, and CD115 antibodies and analyzed by flow cytometry. To assess expression of M-CSFR expression in myeloid cells in the skin, ears from 8 to 13-week-old C57BL/6 mice were collected and frozen in Tissue-Tek OCT embedding medium (Sakura Finetek, Zoeterwoude, Netherlands) and cut into 6 μm-thick sections. Cryosections were fixed and stained as described earlier (33). Optimally titrated goat-anti-rat IgG-Alexa546 and streptavidin-Alexa633 were used to detect unlabeled antibodies and biotinylated antibodies, respectively. Images were acquired using a Leica TCS SP5 confocal microscope.

### Statistical analysis

Statistical analysis between experimental and control groups was carried out using unpaired two-tailed Student’s *t* test (unless stated otherwise) with the Graphpad Prism 5 software package. *P*-values of less than 0.05 were considered significant. Error bars represent mean ± SEM from at least three experiments.

## Results

### MicroRNA expression profiles change during DC development

**Figure 1:**
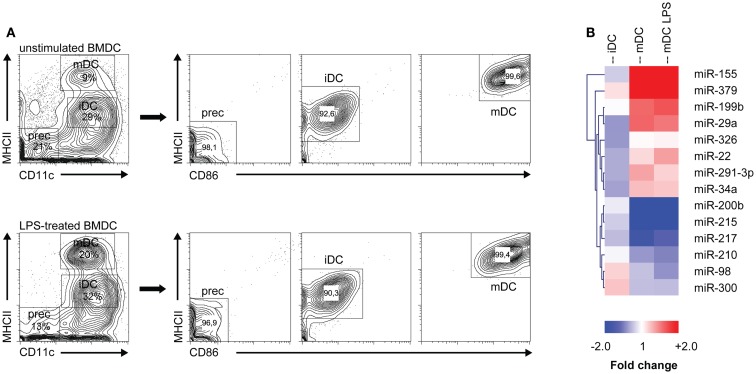
**MicroRNA expression is altered during DC development**. **(A)** Different DC subsets from either unstimulated or LPS-stimulated DC cultures were flow cytometrically sorted according to their differential expression of CD11c, MHC class II, and CD86. Prec, precursor cells; iDC, immature DC; mDC, mature DC. **(B)** Heat map of differentially expressed microRNAs (criteria: false discovery rate ≤ 10%; fold change ≥ 1.5 or ≤−1.5). Each column represents all microRNAs differentially expressed between the DC populations depicted in the heading (iDC, mDC, mDC LPS) and the precursor population. MicroRNA cluster analysis was performed using hierarchical clustering and Euclidean distance measurement.

To investigate which microRNAs are differentially expressed during monocyte-derived DC development *in vitro*, we performed microRNA profiling of distinct DC maturation stages isolated from 7 days GM-CSF-stimulated BM cultures, which were either or not additionally stimulated for 16 h with LPS (i.e., forced vs. spontaneous maturation). Different populations of GM-DC were sorted based on differential expression of maturation markers CD11c, MHC class II, and CD86 (Figure 1A). We then performed profiling of 328 different microRNAs using locked nucleic acid-based microRNA arrays. In total, 14 microRNAs were found to be differentially expressed in iDC to mDC development (Significance Analysis of Microarrays criteria: FDR ≤ 10%; fold change ≥ 1.5 or ≤−1.5, Figure 1B). The changes during spontaneous or LPS-induced maturation appeared to be very similar. Of all microRNAs that were screened, miR-155 showed the most abundant increase, reaching 11- and 17-fold up-regulation in the transition of iDC to mDC or iDC to LPS-mDC, respectively. These findings support data from Ceppi *et al*. (34) who found high levels of miR-155 in human monocyte-derived DC upon LPS stimulation. Conversely, miR-200b and -215 levels were down-regulated approximately threefold in both mDC and LPS-mDC compared to iDC. These results demonstrate that microRNA levels change during DC development *in vitro* and that iDC and mDC are characterized by distinct microRNA expression profiles.

### M-CSFR is a target of miR-22, -34a, and -155, which are up-regulated during final DC maturation

**Figure 2:**
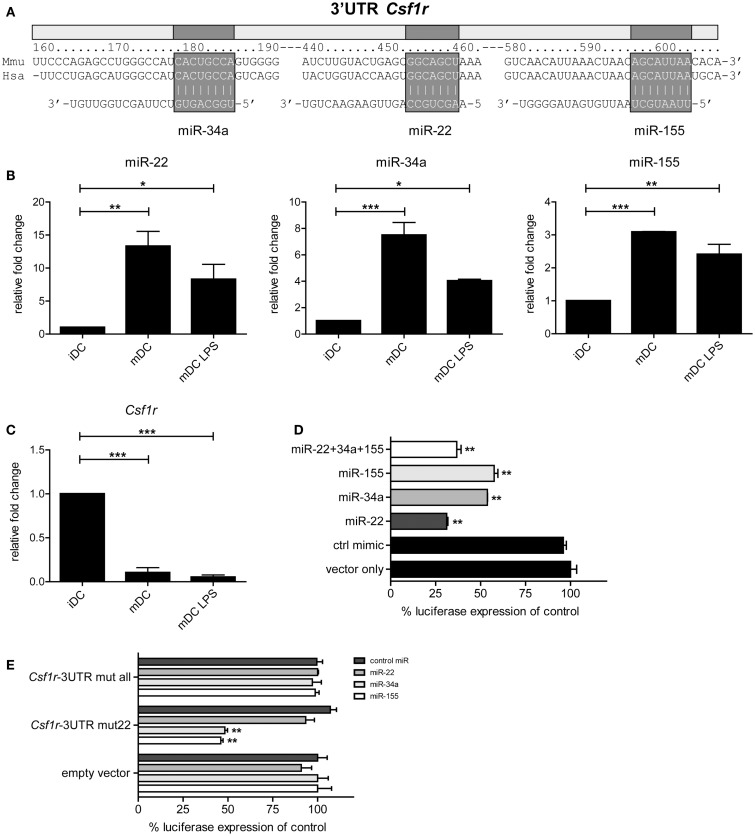
***Csf1r* is a verified target of miR-22, -34a, and -155**. **(A)** Schematic representation of the *Csf1r-*3′UTR showing the predicted conserved microRNA binding sites for miR-22, -34a, and -155. Expression of **(B)** miR-22, -34a, and -155 and **(C)** *Csf1r* mRNA in different sorted DC populations that were used for microRNA profiling, assessed using qPCR. Relative expression changes were calculated using the 2^-ΔΔCt^ method and expressed as fold change over tubulin controls. Data are expressed as means ± SEM from three experiments; *p < 0.05, **p < 0.01, ***p < 0.001 compared with iDC control. **(D,E)** The full-length *Csf1r-*3′UTR was cloned downstream a *Renilla luciferase* (RLuc) gene (psiCHECK-2 vector). HEK293T cells were transiently transfected with the psiCHECK-2 reporter vector containing **(D)** the wild-type mouse *Csf1r*-3′UTR or **(E)** a mutant *Csf1r*-3′UTR harboring three nucleotide point mutations in each seed region of the corresponding miR-22, -34a, and miR-155 binding sites (*Csf1r*-3′UTR mut all), or in the miR-22 site only (*Csf1r*-3′UTR mut22). The original psiCHECK-2 vector (containing no 3′UTR insert) was used as a control (empty vector). Cells were co-transfected with the hairpin precursors of miR-22, miR-34a, miR-155, or control oligonucleotide, all at a final concentration of 50 nM. A ubiquitously expressed firefly luciferase gene, also encoded by the vector, was used to normalize transfection efficiency. Luciferase activity was measured 48 h after transfection. Data are expressed as means ± SEM from three experiments.

To investigate which developmental genes are regulated by microRNAs in the final maturation step from iDC to mDC we compared the list of differentially expressed microRNAs with one compiled for genes known to be involved in the development of myeloid cells (DC/macrophage/neutrophil) as listed in the KEGG (Kyoto Encyclopedia of Genes and Genomes) database (35) (KEGG entry: mmu04640; Hematopoietic cell lineage – *Mus musculus*). Using the Targetscan algorithm (36), we identified that 10 out of these 30 genes have predicted microRNA target sites in their 3′UTR conserved across mammals. Subsequently, we compared these 10 genes to our microRNA profiling data (Figure 1B), and found that only *Csf1r*, the gene encoding M-CSFR, and *Kitl* (SCF, stem cell factor) were potentially regulated by microRNAs differentially expressed in GM-DC. From these, M-CSFR was the most likely target for microRNA regulation in iDC to mDC transition as three out of four conserved predicted binding sites in the 3′UTR of *Csf1r* mRNA were targeted by differentially expressed microRNAs, i.e., miR-22, -34a, and -155 (Figure 2A). Other microRNA target prediction algorithms (incl. PicTar, EIMMo) confirmed the miR-22, -34a, and -155 binding sites in both human and mouse *Csf1r-*3′UTR (not shown). Interestingly, expression of all three microRNAs was up-regulated upon iDC to (LPS-) mDC transition in our array, which we could confirm by quantitative RT-PCR (Figure 2B). In accordance, we found a strong down-regulation of *Csf1r* mRNA levels in mDC compared to iDC (Figure 2C).

To test whether miR-22, -34a, and -155 actually can regulate *Csf1r* expression through direct 3′UTR interactions, we cloned the complete 3′UTR of *Csf1r* into the psiCHECK-2 reporter vector downstream the coding sequence of *Renilla luciferase*. This vector, which also encoded firefly luciferase as an internal control, was transfected into HEK293T cells. When miR-155- or miR-34a precursor microRNAs were co-transfected we observed that *Renilla luciferase* expression was repressed approximately 50% compared to controls (Figure 2D). Co-transfection of miR-22 precursor inhibited *Renilla luciferase* expression even almost 75%. The combination of all three microRNAs did not further down-regulate luciferase expression. These findings confirm and extend the work of O’Connell et al. (37) and Lu *et al*. (24) who have shown that *Csf1r* is a validated target of miR-155. To verify whether the repressive effects were microRNA-specific, we repeated these experiments with *Csf1r-*3′UTR reporter constructs harboring point mutations in all three (miR-22, -34a, and -155) microRNA binding sites or in just one (miR-22) microRNA binding site. Indeed, no repressive effects of microRNAs could be observed when mutants were tested containing alterations in all binding sites, indicating the specificity of microRNA targeting (Figure 2E). Mutation of the miR-22 binding site alone (but not the miR-34a and -155 binding sites) completely impaired miR-22-mediated *Csf1r-*3′UTR repression, whereas the repressive effects of miR-34a and -155 remained unaffected, with similar levels of repression as in our initial experiments with the wild-type mouse *Csf1r-*3′UTR (Figure 2E).

Taken together, these data demonstrate that miR-22, -34a, and -155 are up-regulated in the final step of iDC to mDC maturation and that all three microRNAs can directly regulate *Csf1r* expression by targeting its mRNA via 3′UTR interactions.

### M-CSFR protein expression is down-regulated in the final phase of DC maturation

**Figure 3:**
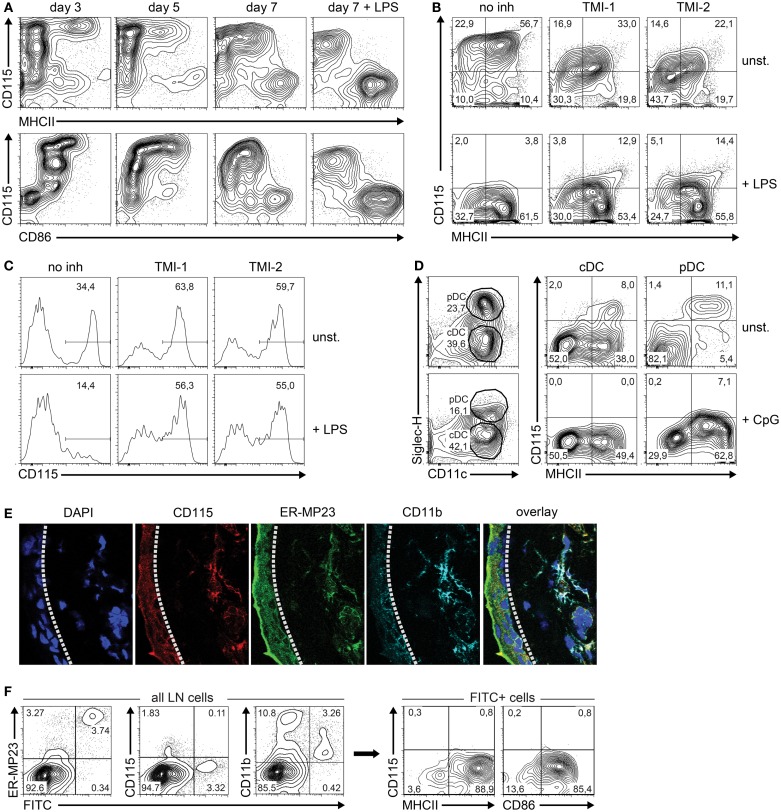
**M-CSFR protein expression is regulated during DC maturation**. **(A)** Flow cytometry contour plots show cell surface M-CSFR (CD115) protein expression over time plotted against the DC maturation markers MHC class II and CD86 in GM-CSF-stimulated DC cultures. LPS (100 ng/ml) was added on day 6 as a positive control to induce DC maturation. **(B)** Effect of TACE inhibition by TMI-1 or -2 on M-CSFR/CD115 expression during the final 24 h of GM-DC culture, in presence or absence of LPS-stimulated maturation. **(C)** Effect of TACE inhibition during overnight culture of fresh BM on spontaneous (top panel) and LPS-induced (lower panel) down-regulation of M-CSFR/CD115 expression on BM monocytes, identified by gating of CD11b^+^Ly-6C^hi^Ly-6G^neg^ cells (not shown). **(D)** Detection of M-CSFR/CD115 expression on mature and immature *in vitro*-derived FL-cDC and pDC. Mouse BM cells were cultured for 8 days with Flt3L and surface marker expression was analyzed using flow cytometry. CpG was added on day 7 to induce DC maturation. Viable DC were gated using side scatter and negative DAPI staining as criteria (not shown). Next, differential SiglecH and CD11c expression was used to distinguish between pDC (CD11c^+^SiglecH^+^) and cDC (CD11c^+^SiglecH^–^). CD115 expression was plotted against the DC maturation marker MHC class II for both cDC and pDC. Results are shown of a representative experiment of three with similar outcome. **(E)** Macrophages and M-CSFR-dependent immature DC in sections of mouse ear skin dermis were identified by CD115 (red), mMGL/CD301 (green), CD11b (cyan), and DAPI (blue), and detected by confocal microscopy. The dashed line indicates the border of the epidermis (left) and dermis (right). **(F)** Analysis of skin-derived FITC^+^ cells migrated to skin-draining LN. Mice were skin-painted on shaved back with a 1% FITC solution. After 24 h, skin-draining LN were isolated, processed into single-cell suspensions by mechanical means only and analyzed using flow cytometry. FITC^+^ cells emigrated from the skin were found to represent primarily CD11c^+^MHCII^hi^CD86^hi^ cells lacking CD115 expression. Results of **(E,F)** are shown of a representative experiment of three with similar outcome.

Based on the differential expression of *Csf1r*-regulating microRNAs and their *Csf1r* target mRNA, we predicted that M-CSFR protein expression decreased during the transition of iDC to mDC. Therefore, we followed M-CSFR expression in GM-CSF-stimulated BM cultures over time by flow cytometry. To induce final maturation, part of the cultures was treated with LPS on day 6. Indeed, M-CSFR expression was rapidly down-regulated during both spontaneous and LPS-induced transition of iDC to mDC, accompanied by the acquisition of high level MHC class II and CD86 expression (Figure 3A).

Although our results suggest that microRNAs are responsible for the observed down-regulation of M-CSFR expression, we cannot exclude other mechanisms that might also be involved in this process. In particular, LPS is known to stimulate activation of the transmembrane protease TNF-α-converting enzyme (TACE), which cleaves M-CSFR from the surface (38). To study a putative contribution of TACE-mediated M-CSFR cleavage to decreasing expression during final maturation, GM-DC were incubated with and without TACE inhibitors TMI-1 and -2 (28, 29) before enforcing DC maturation with LPS during overnight culture. We found that M-CSFR levels decreased significantly in LPS-stimulated DC compared to unstimulated cells, despite inhibition of TACE (Figure 3B). TMI-1 and -2 treatment of GM-DC by itself already caused a decrease in M-CSFR expression, which was not caused by decreased cell viability (not shown). The efficacy of the TACE inhibitors under these conditions is indicated by the inhibition of spontaneous and LPS-induced decrease of membrane-bound M-CSFR on BM monocytes [compare left panels in Figure 3C to approximately 80% M-CSFR-expressing BM monocytes upon isolation (39)] (Figure 3C). Collectively, these data suggest that TACE-mediated shedding does not play a major role in reducing M-CSFR expression on maturing GM-DC.

To investigate whether down-regulation of surface M-CSFR expression is restricted to maturing GM-DC, we cultured freshly isolated BM in the presence of Flt3L to generate cDC (FL-cDC) and pDC and assessed surface M-CSFR levels by flow cytometry. We observed that only a minor subset of CD11c^+^SiglecH^−^ FL-cDC and CD11c^+^SiglecH^+^ pDC expressed M-CSFR (Figure 3D). Although enforced maturation with CpG in these cultures led to a further reduction in M-CSFR levels, we conclude that M-CSFR expression by iDC and down-regulation during their maturation is not a universal characteristic of Flt3L-generated cDC and pDC *in vitro*.

Next, we asked whether M-CSFR expression is regulated in DC *in vivo* as well. To that end, we focused on DC present in the skin, and assessed their M-CSFR expression *in situ* and after stimulated migration to skin-draining LN. The skin harbors various cells of myeloid origin, such as Langerhans cells in the epidermis, and macrophages/iDC in the dermis (1, 33, 40). Both epidermal and dermal populations of cells are able to incorporate skin-applied antigens and migrate subsequently to the draining LN, where they appear as phenotypically mDC (33, 41). Although flow cytometric analysis is the preferred method to quantify protein expression in mixed cell populations, we observed that the sample preparation of mouse skin tissue into single-cell suspensions using enzymatic digestion did not allow the use of flow cytometry due to enzymatic cleavage of surface-bound M-CSFR (not shown). Therefore, we analyzed M-CSFR expression by skin mononuclear phagocytes *in situ* by less sensitive confocal microscopy of tissue sections. We labeled mouse ear skin cryosections with CD115 – an antibody directed against M-CSFR – together with mMGL (ER-MP23) and CD11b antibodies to identify all dermal macrophages and iDC of the partially M-CSFR-dependent CD103^neg^ subset (7) and analyzed expression of these markers (Figure 3E). Cells that stain positively for the C-type lectin mMGL (CD301) are dermal macrophages and iDC that migrate to draining LN upon antigen uptake (33, 42). The images demonstrate that CD115 co-localizes with CD11b in the skin epidermis as well as with CD11b and mMGL in the dermis of the mouse ear. Similar observations were made in sections taken from mouse back skin (not shown). This indicates that both epidermal Langerhans cells and dermal mononuclear phagocytes uniformly express M-CSFR *in situ*, in agreement with previous findings by Hume *et al*. at the mRNA level (43).

To assess whether these CD115-positive mononuclear phagocytes in the skin down-regulate expression upon maturation during migration to draining LN, we skin-painted FITC onto shaved back skin of mice and analyzed the phenotype of the FITC^+^ cells that had emigrated from the skin to skin-draining LN 24 h later (Figure 3F). The majority of LN-immigrating, FITC-transporting cells from the skin express mMGL (ER-MP23) at a high level, reminiscent of their dermal origin. These findings are in agreement with our previous results (33) and with those of Irimura and colleagues (42). The skin-derived cells, expressing CD115 *in situ*, have lost CD115 expression upon LN arrival. Furthermore, FITC-positive LN cells have retained CD11b at an intermediate or low level, related to a DC nature, rather than the high level CD11b^+^ myelomonocytic cells, which are FITC-negative and partially M-CSFR^+^ (not shown). In agreement with their DC identity, gated FITC-positive LN cells show high levels of MHC class II and CD86 expression.

Together, our data demonstrate that M-CSFR protein is expressed by DC at the precursor and iDC stage, but down-regulated in the final stage of DC maturation. We show this during *in vitro* maturation of DC derived from BM precursors stimulated with GM-CSF, and our *in vivo* findings on maturation of connective tissue DC that migrate to skin-draining LN support this notion. In agreement with this, Cheong *et al*. have shown that monocytes down-regulate M-CSFR expression upon maturation to inflammatory LN DC *in vivo* (44).

### Inhibition of miR-22, -34a, and -155 dysregulates M-CSFR expression and blocks DC maturation

**Figure 4:**
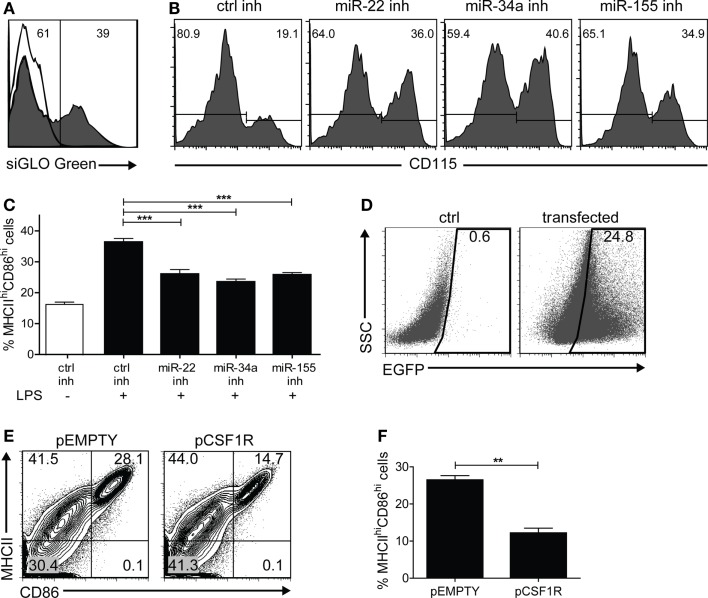
***In vitro* inhibition of miR-155/-34a/-22 prevents LPS-induced M-CSFR down-regulation and DC maturation**. BM cultures stimulated with GM-CSF were transfected with different microRNA oligonucleotide inhibitors on day 4, together with siGLO Cy3-labeled non-targeting anti-miR oligonucleotides at a ratio of 5:1, and exposed to LPS on day 6. Cell surface marker expression was analyzed on day 7. **(A)** Only successfully transfected cells (siGLO^+^) were selected for analysis. The open histogram represents non-transfected cells. **(B)** Inhibition of miR-22, -34a, and -155 prevented LPS-induced CD115 down-regulation compared to control inhibitor alone. Results are shown of a representative experiment of five with similar results. **(C)** Frequency of mDC (MHCII^hi^CD86^hi^) in cultures stimulated or not with LPS and transfected with control inhibitor or miR-22, -34a, or -155 inhibitor. Data are expressed as mean ± SEM from five experiments; ***p < 0.0001. **(D)** BM cultures stimulated with GM-CSF were nucleofected on day 6 of culture with an expression vector encoding *Csf1r* (pCSF1R) or the empty backbone vector (pEMPTY) together with an EGFP expression vector in a 4:1 ratio to assess transfection efficiency. LPS was added on day 8 to enforce DC maturation. In this experiment, transfection led to detectable EGFP expression in 24% of the cells. 7AAD staining confirmed that both vectors used did not cause significant differences in cell viability as a result of transfection (data not shown). **(E,F)** Cell surface marker expression of *Csf1r*-transfected EGFP^+^ cells was analyzed on day 9 using flow cytometry. Contour plots of transfected cells **(E)** and quantitation of viable MHCII^hi^CD86^hi^EGFP^+^ mDC **(F)** after overnight LPS exposure. Data are expressed as mean ± SEM from three experiments; **p = 0.0013.

At this point, it is reasonable to hypothesize that up-regulation of miR-22, -34a, and -155 is causally involved with *Csf1r* down-regulation. To substantiate this, we investigated whether M-CSFR protein down-regulation in maturing DC *in vitro* could be prevented by inhibiting miR-22, -34a, and -155 using microRNA inhibitor oligonucleotides. First, we used siGLO Cy3-labeled non-targeting anti-miR oligonucleotides to evaluate transfection efficiency, generally varying between 30 and 40% (Figure 4A). Then we inhibited microRNA-22, -34a, or -155 on day 4 of GM-DC culture and observed that M-CSFR down-regulation upon LPS-induced DC maturation at day 7 was significantly prevented (Figure 4B), demonstrating that miR-22, -34a, and -155 regulate M-CSFR expression in DC in the final stage of maturation. Interestingly, in these experiments we observed that not only down-regulation of M-CSFR was reduced on cells treated miR-22, -34a, or -155 inhibitors, compared to the cells transfected with control inhibitor, but that also the frequency of cells with a mDC phenotype (CD11c^+^MHCII^hi^CD86^hi^) was significantly lower (*p* < 0.0001) (Figure 4C). This suggests that these microRNAs are directly involved with the final step in DC maturation. To investigate whether microRNA-mediated M-CSFR down-regulation was functionally involved in this step, we enforced *Csf1r* expression in developing GM-DC using an expression vector containing the complete ORF of mouse *Csf1r*. Cells were co-transfected with an EGFP expression vector at a 1:4 ratio (EGFP: *Csfr1*) to enable selection for successfully transfected cells (Figure 4D). In these experiments we found that enforced *Csf1r* expression significantly (*p* = 0.0013) impaired up-regulation of DC maturation markers MHC class II and CD86 upon stimulation with LPS (Figures 4E,F).

In summary, these results show that *in vitro* inhibition of miR-22, -34a, and -155 reduces LPS-induced M-CSFR down-regulation in GM-DC. Moreover, our findings suggest that down-regulation of M-CSFR in DC is required for full DC maturation.

## Discussion

**Figure 5:**
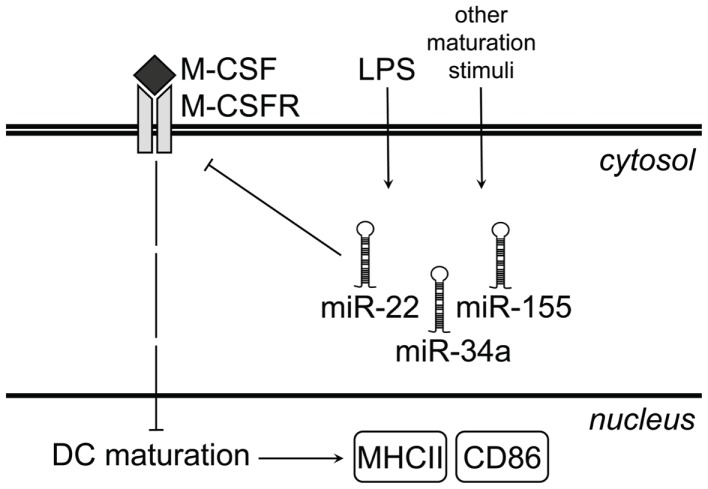
**Proposed model for microRNA-mediated regulation of M-CSFR upon LPS-induced GM-DC maturation**. Under steady-state conditions, immature GM-DC express high levels of M-CSFR. As a result of M-CSFR signaling, expression of maturation markers such as MHC class II and CD86 remains low. LPS stimulation of iDC or other maturation-inducing stimuli up-regulate miR-22, -34a, and -155, which bind to *Csf1r* mRNA and prevent translation of M-CSFR protein. This results in down-regulation of M-CSFR protein at the cell surface and allows up-regulation of both MHC class II and CD86.

In this study we provide evidence that microRNAs play an important role in mouse GM-DC development by down-regulating M-CSFR expression in the final maturation step in which DC acquire the mature MHC class II^hi^ CD86^hi^ phenotype (Figure 5). We show that the transition from CD11c^+^ MHC class II^med^ CD86^−/lo^ iDC to CD11c^+^ MHC class II^hi^ CD86^hi^ mDC coincides with an up-regulation of some microRNAs and down-regulation of others. Noteworthy in this respect is that spontaneously *in vitro* matured mDC and LPS-induced mDC show remarkably similar microRNA expression profiles. This suggests that spontaneous, GM-CSF-induced and LPS-induced maturation proceed along similar routes, at least on a microRNA level.

Based on our profiling results, we have identified *Csf1r* as a primary target of microRNA-mediated regulation. The role of the *Csf1r* gene in myeloid cell development has been studied at several levels, but its function in DC development has remained underexposed (45). MacDonald *et al*. have demonstrated in the MacGreen mouse model, in which the *Csf1r* promoter directs the expression of EGFP, that the *Csf1r* gene is generally expressed by DC *in vivo* (6). Moreover, the significant reduction of DC in mice lacking *Csf1r* indicates that this gene is required for optimal expansion of DC (5–7). Although the MacGreen mouse model shows transcriptional activity of the *Csf1r* promoter throughout DC differentiation in GM-CSF-driven BM cultures, it does not necessarily indicate presence of the M-CSFR protein product (6). Our data demonstrate that *Csf1r* expression is actively down-regulated in the final phase of DC maturation at both the mRNA and protein level via microRNAs.

MicroRNAs are involved in the regulation of M-CSFR expression both directly and indirectly during myeloid cell differentiation. MicroRNAs 17-5p, -20a, and -106 regulate M-CSFR indirectly through targeting of the AML1/Runx1 transcription factor (46). In early myeloid precursor cells these microRNAs suppress AML1/Runx1 protein expression, leading to limited *Csf1r* transcription and inhibition of monocytic differentiation and maturation. In our profiling experiments, we observed no major changes in miR-17-5p, -20a, or -106 expression during DC development, suggesting that this regulation does not play an important role in later stages. O’Connell and colleagues have previously validated a significant number of targets of miR-155, including *Csf1r* (37), a finding that was recently confirmed by Lu *et al*. (24). However, these studies did not address the question whether microRNA-mediated regulation of *Csf1r* influences myeloid cell development. Our data provide evidence that final DC differentiation requires microRNA-mediated regulation of *Csf1r*. Additionally, we show here that miR-22 and miR-34a also directly target *Csf1r* and are up-regulated upon final DC maturation.

Tight regulation of *Csf1r* expression is essential for proper myeloid cell development and occurs at several levels. At a transcriptional level, the *Csf1r* gene is transactivated by several myeloid transcription factors, including PU.1, Runx1, C/EBPα, and several Ets family members through binding to its TATA box-deficient promoter located ∼300 bp upstream of the transcriptional start site (47–49). A highly conserved intronic enhancer named FIRE is located in the first intron downstream of the *Csf1r* promoter and contains additional Sp1 and Egr-2 binding motifs (50, 51). At the protein level, membrane-bound M-CSFR dimerizes after binding of its ligand M-CSF to macrophages, resulting in a number of modifications to the cell surface receptor including tyrosine/serine phosphorylation, ubiquitination, and subsequent internalization of the M-CSF/M-CSFR complex before its intralysosomal degradation (52). Additionally, various pro-inflammatory stimuli cause enzymatic cleavage of the M-CSFR ectodomain by TNFα-converting enzyme (TACE)-mediated shedding *in vitro* (38, 53, 54). However, *in vivo* down-regulation of surface M-CSFR expression under inflammatory conditions can also be TACE-independent, since TACE was not responsible for absence of M-CSFR expression from monocytes generated during severe *L. monocytogenes* infection (39). Our current experiments indicated that TACE also did not play a major role in M-CSFR down-regulation in final GM-DC maturation *in vitro*. Other post-translational modifications to the M-CSFR protein, such as ubiquitination or sumoylation, probably also contribute to M-CSFR instability. For instance, binding of M-CSF to M-CSFR induces conformational changes to the receptor leading to M-CSFR ubiquitination and subsequently altered protein half-life (55, 56).

Here, we have shown that an additional level of *Csf1r* expression regulation occurs, on top of transcriptional and post-translational control, through the action of microRNAs miR-22, -34a, and -155. Interestingly, two studies have indicated that DC from *Mir155^−/−^* mice show impaired expression of maturation markers after LPS stimulation and have impaired T cell stimulatory capacity (57, 58). In contrast, Lu *et al*. reported no such differences in miR-155-deficient DC after LPS stimulation (24), but the levels of LPS used to induce DC maturation in the latter study were at least 10-fold higher than those used in our and other studies (57, 58). Therefore, it is possible that hyper-stimulation by LPS annuls the attenuated DC maturation phenotype caused by miR-155 deletion. Direct comparison of M-CSFR-regulating microRNA levels and M-CSFR mRNA and protein expression in LPS- vs. spontaneously matured DC (Figures 2B,C, and 3A) indicates that differences at microRNA level, although important, do not provide a full explanation for differences at mRNA and protein level, and therefore post-translational mechanisms described above probably play an important additional role. Together, this shows that post-transcriptional microRNA-mediated regulation of *Csf1r* mRNA expression complements its transcriptional regulation, in line with the notion that *Csf1r* is stringently regulated during myelopoiesis.

To address the role of M-CSFR during final GM-DC maturation, we inhibited its down-regulation via microRNA inhibitors or transiently over-expressed *Csf1r* in GM-DC. Both approaches resulted in a significant inhibition of LPS-induced final DC maturation. Interestingly, mice lacking the *Csf1r* gene show major alterations in different subsets of DC, where especially the CD11b^+^ CD103^−^ subset is affected (6, 7). Therefore, both absence of *Csf1r* as well as enforced expression lead to aberrant DC development. This underscores that regulation of the levels of this receptor throughout DC differentiation is crucial. Our analysis of M-CSFR expression by skin and LN DC suggests that this regulation also occurs during *in vivo* maturation of DC. In agreement with these findings, Cheong and colleagues have shown that mouse blood monocytes, which are recruited to peripheral LN after i.v. injection of Gram-negative bacteria or LPS, also lose their M-CSFR expression upon *in vivo* differentiation to DC in the draining LN (44). It is tempting to speculate that especially expression of miR-155, stimulated by the inflammatory conditions (59), is involved with M-CSFR down-regulation in these infection-induced monocytes and monocyte-derived DC. The importance of post-transcriptional regulation of *Csf1r* is further supported by Sasmono *et al*. who have found that mouse neutrophilic granulocytes contain significant amounts of *Csf1r* mRNA, but do not express the corresponding protein product (60). Involvement of microRNAs in this regulation has not been shown, however.

How might M-CSFR-mediated inhibition of DC differentiation operate at the molecular level? Although speculative, a possible scenario is that M-CSFR triggering leads to sustained activity of the PI-3K/Akt pathway (52). This then inhibits LPS-induced activation of p38 MAPK, JNK and NF-κB which are important for the expression of mDC characteristics such as MHC class II and CD86 (61, 62). This view is supported by a study showing that GM-DC from mice lacking SHIP, a negative regulator of the PI-3K pathway, have an immature phenotype and mature poorly in response to LPS (63). Notably, this apparent block in DC maturation could be inverted by treating these cells with PI-3K inhibitors LY294002 or Wortmannin, indicating that PI-3K is a negative regulator of DC maturation. Therefore, M-CSFR-mediated activation of the PI-3K pathway might block final LPS-induced DC maturation. Additionally, M-CSFR-signaling elevates levels of c-Fos (64), whereas miR-155-mediated down-regulation of c-Fos was recently shown to be involved with DC maturation and function (58). It should be stressed, however, that M-CSFR signaling involves a variety of pathways, including those mediated by Ras, Jak/STAT, or β-catenin. Collectively, these downstream mechanisms are responsible for the final outcome of M-CSFR signaling in macrophages (52, 65). Which of these pathways is responsible for inhibition of DC maturation by M-CSFR activity remains to be demonstrated.

In summary, we have elucidated a previously unknown molecular mechanism regulating the final step in monocyte-derived DC maturation, which acts upstream of M-CSFR signaling and where the receptor itself is subject to microRNA-mediated control. Our work demonstrates that decreasing M-CSFR signaling contributes to enable final GM-DC maturation.

## Conflict of Interest Statement

The authors declare that the research was conducted in the absence of any commercial or financial relationships that could be construed as a potential conflict of interest.
